# Supplementary figures and images for: Physiological and Genetic Dissection of Sucrose Inputs to the Arabidopsis thaliana Circadian System
Source: Genes (Basel). 2019 May 2;10(5):334. doi: 10.3390/genes10050334 (PMC6563356; doi:10.3390/genes10050334)

# Supplementary Figure 1

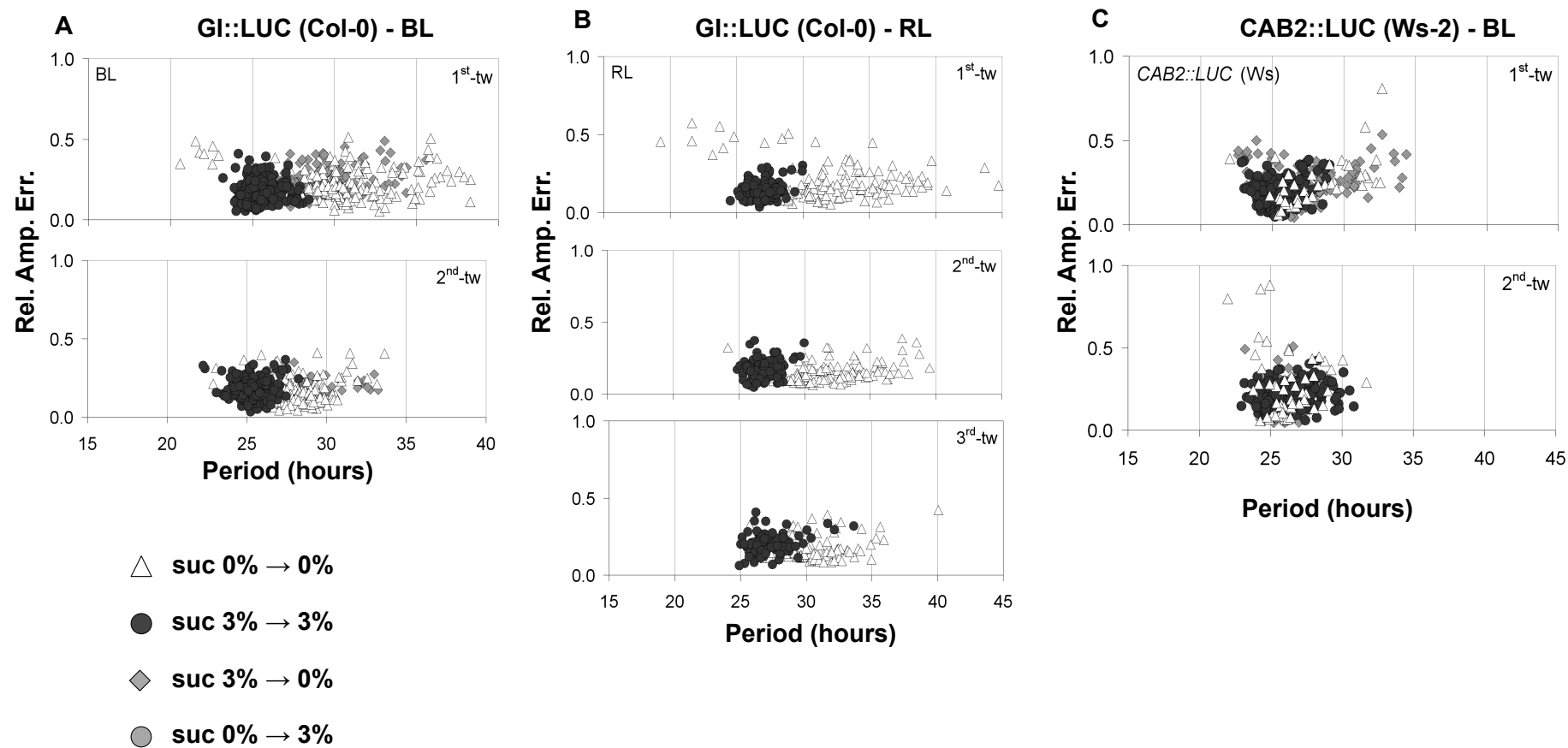

Supplementary Figure 2

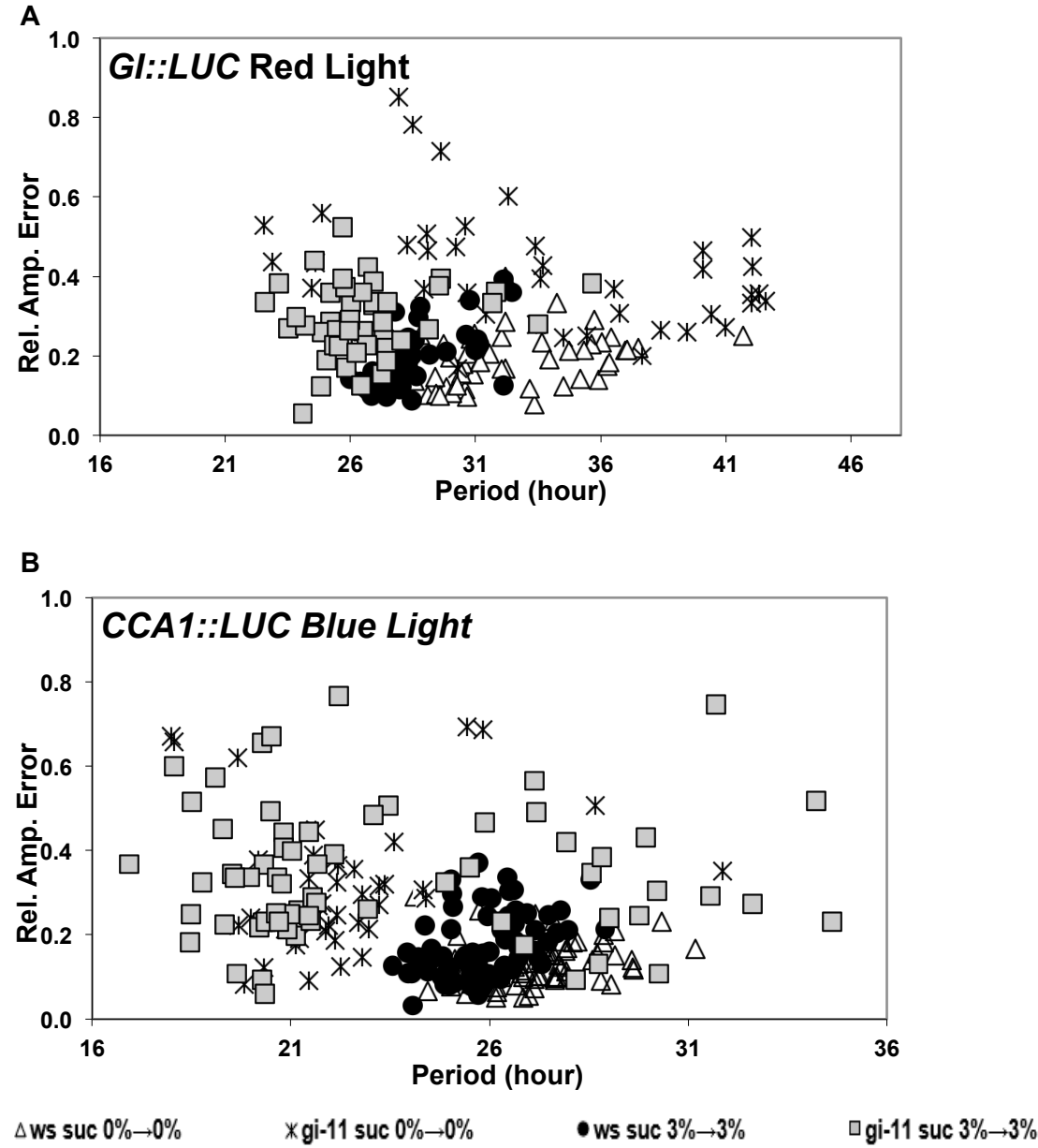

Supplement: Supplementary file 1 [file genes-10-00334-s001.pdf]
